# Foveal-Onset Acute Retinal Necrosis Associated With Varicella-Zoster Virus and Delayed Peripheral Retinitis: A Case Report

**DOI:** 10.7759/cureus.113324

**Published:** 2026-07-24

**Authors:** Takumi Tsuchikawa, Masayuki Inuzuka, Mami Goda, Kiyofumi Mochizuki, Toshio Hisatomi

**Affiliations:** 1 Ophthalmology, Gifu University Graduate School of Medicine, Gifu, JPN

**Keywords:** acute retinal necrosis, aqueous humor, foveal lesion, ocular hypertension, optical coherence tomography, polymerase chain reaction, varicella-zoster virus

## Abstract

Acute retinal necrosis (ARN) usually begins in the peripheral retina, but atypical cases may initially involve the posterior pole. A 63-year-old man with no evidence of immunodeficiency presented with decreased vision and ocular hypertension in the left eye. The earliest optical coherence tomography (OCT) image showed a lesion centered on the fovea. At a subsequent examination, a whitish foveal lesion was associated with marked hyperreflectivity involving nearly the entire inner retina, posterior shadowing, and ellipsoid zone disruption. Dilated peripheral retinal examination and widefield fundus photography at the referring hospital showed no visible peripheral necrotizing retinitis; scleral depression was not performed. OCT angiography showed no detectable perfusion abnormality within the acquired 6 x 6-mm macular scan in either the superficial or deep retinal slab. Fine keratic precipitates, increased aqueous flare, and ocular hypertension raised concern for herpetic retinitis, and oral valacyclovir was started. At the first visit to our hospital, aqueous humor was collected while no peripheral lesion was visible. Four days later, an approximately four-disc-diameter inferotemporal lesion with vascular sheathing, punctate hemorrhages, and vitreous opacity appeared. On the same day, the 24-target multiplex PCR result from the aqueous sample collected at the first visit became available and was positive only for Varicella-zoster virus (VZV) DNA. Antiviral therapy with systemic corticosteroids led to regression of the retinal lesions. At 42 weeks, best-corrected visual acuity had improved to 0.6, with no retinal detachment or recurrence. This case suggests that VZV-associated ARN may initially present as an isolated foveal lesion. The OCT morphology was not typical of paracentral acute middle maculopathy because the hyperreflectivity was not confined to the middle retinal layers.

## Introduction

Acute retinal necrosis (ARN) is a sight-threatening necrotizing herpetic retinitis that most often occurs in individuals without recognized immunodeficiency. The classic clinical criteria proposed by the American Uveitis Society emphasize one or more well-demarcated areas of peripheral retinal necrosis, rapid progression without antiviral therapy, circumferential spread, occlusive retinal vasculopathy, and prominent intraocular inflammation [1]. More recently, the Standardization of Uveitis Nomenclature (SUN) Working Group proposed classification criteria incorporating peripheral necrotizing retinitis together with virologic or characteristic clinical evidence [2]. Delayed recognition and treatment increase the risk of severe visual loss and rhegmatogenous retinal detachment; therefore, prompt antiviral therapy is essential [3].

Although Varicella-zoster virus (VZV)-associated ARN is well characterized, posterior pole or macular involvement may occasionally precede the typical peripheral findings [4-9]. Such cases are difficult to diagnose because a solitary macular white lesion can initially suggest an ischemic or inflammatory maculopathy. Classic paracentral acute middle maculopathy (PAMM), however, is characterized on optical coherence tomography (OCT) by a band-like hyperreflective lesion centered at the outer plexiform layer and inner nuclear layer, followed by thinning of the inner nuclear layer [10]. We report a case in which the earliest documented lesion was centered on the fovea and the subsequent broad retinal hyperreflectivity and accompanying inflammatory signs were more consistent with early necrotizing viral retinitis than with PAMM.

An earlier version of this case report was posted as a preprint on Research Square [11]. The present manuscript has been substantially revised, particularly with regard to the interpretation of the initial OCT findings and the clinical significance of the foveal-onset presentation.

## Case presentation

The day of presentation to our hospital was defined as hospital day 0. A 63-year-old man developed decreased visual acuity in the left eye approximately 17 days before presentation to our hospital. He had no history of immunodeficiency, diabetes mellitus, or malignancy. HIV serology was negative. HbA1c was 6.1%, physical examination and laboratory testing did not suggest collagen vascular disease, and systemic computed tomography showed no evidence of malignancy. No evidence of immunodeficiency was identified.

**Figure 1:**
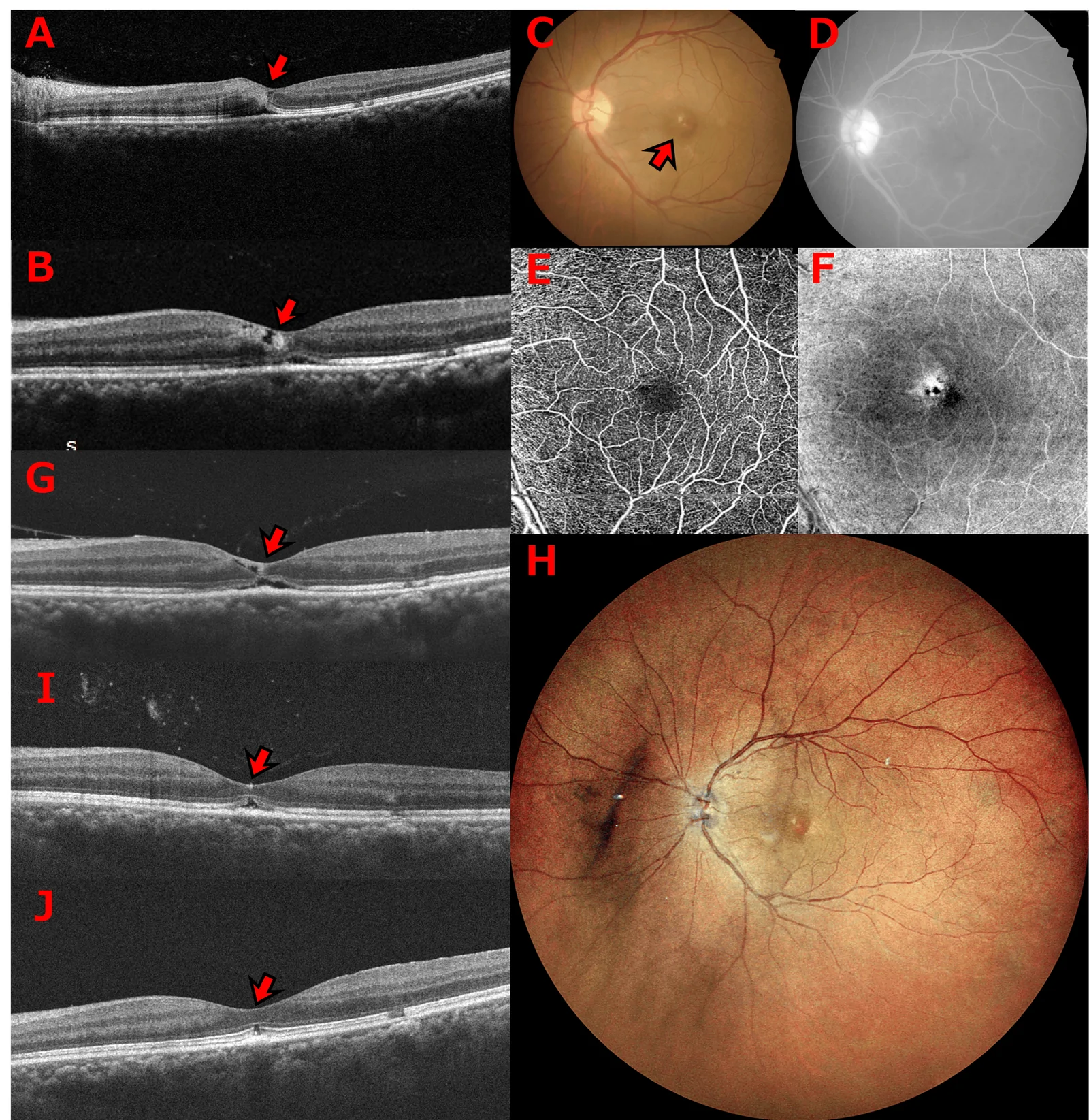
Sequential multimodal imaging of foveal-onset acute retinal necrosis (ARN) associated with Varicella-zoster virus (VZV) (A) Optical coherence tomography (OCT) at the first local ophthalmology clinic shows a lesion centered on the fovea. (B-F) Findings at the referring hospital three days before presentation to our hospital. (B) OCT demonstrates marked hyperreflectivity involving nearly the entire inner retina at the fovea, with posterior shadowing and ellipsoid zone disruption. (C) Color fundus photography shows a whitish lesion localized to the foveal center. (D) Fluorescein angiography shows mild macular hyperfluorescence without apparent retinal vascular occlusion or retinal vasculitis. (E) The superficial retinal slab of a 6 x 6-mm macular OCT angiography scan shows no detectable perfusion abnormality within the scanned area. (F) The deep retinal slab also shows no detectable perfusion abnormality within the scanned area. (G, H) Findings at the first visit to our hospital. (G) OCT shows persistent broad inner retinal hyperreflectivity and foveal ellipsoid zone disruption. (H) Color fundus photography shows the foveal lesion without additional lesions in the imaged posterior pole. (I) Three weeks after treatment initiation, OCT shows regression of the inner retinal hyperreflectivity and partial restoration of the ellipsoid zone outside the foveal center. (J) At the final follow-up, 42 weeks after treatment initiation, OCT shows stable retinal architecture and preservation of the ellipsoid zone outside the foveal center. Red arrows indicate the foveal lesion or the corresponding foveal OCT abnormality at each time point.

**Figure 2:**
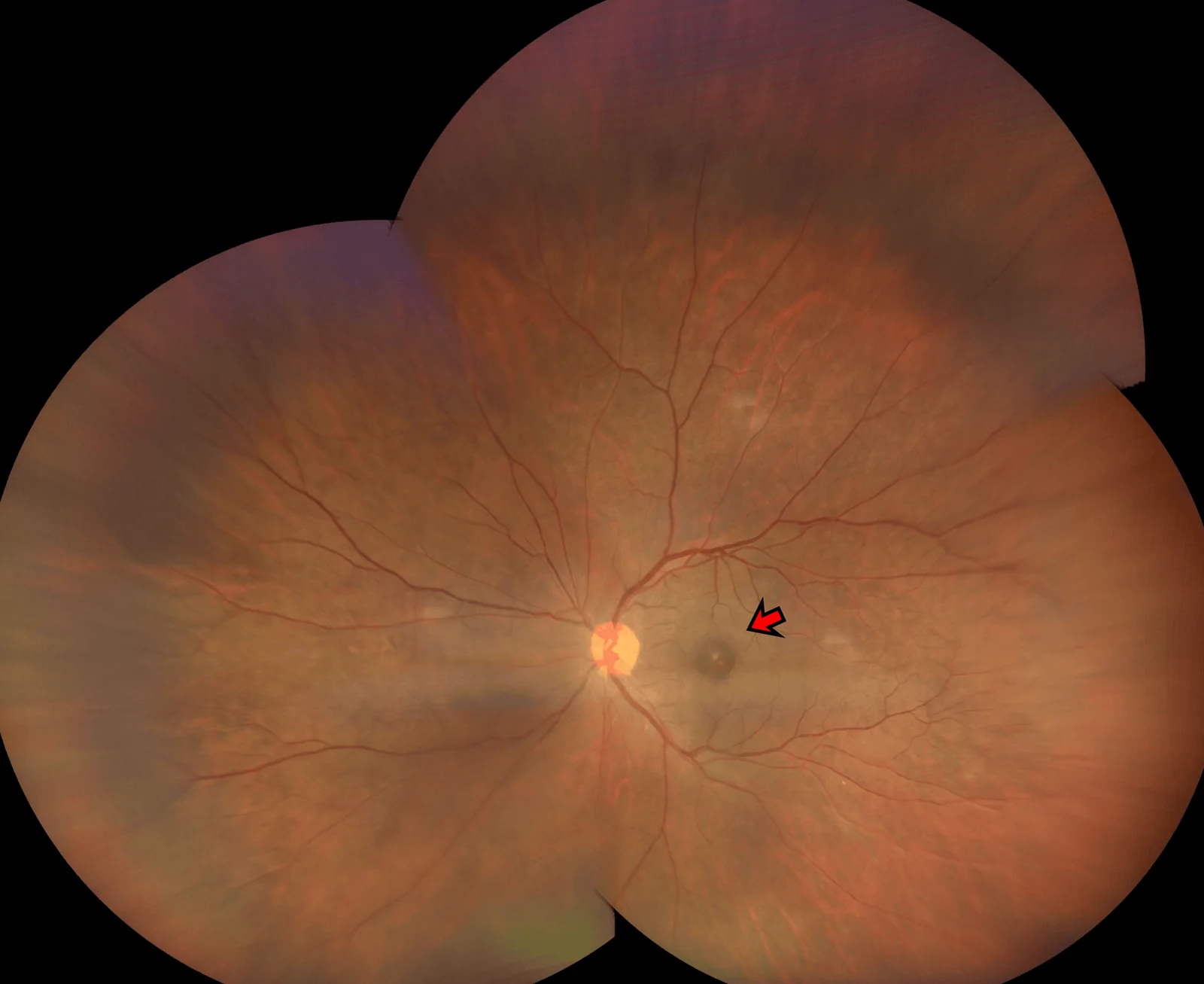
Widefield color fundus montage obtained at the referring hospital three days before presentation to our hospital The image shows the foveal lesion and no visible peripheral necrotizing retinitis within the photographed field. The red arrow indicates the foveal lesion. Scleral depression was not performed.

Thirteen days before presentation to our hospital, he visited a local ophthalmology clinic, where OCT obtained using a Topcon system showed a lesion centered on the fovea (Figure 1A). The exact Topcon model and scan protocol were not available in the record. Ten days later, three days before presentation to our hospital, he was referred to the ophthalmology department of another hospital because macular degeneration was suspected. Best-corrected visual acuity in the left eye was 0.2, and intraocular pressure (IOP) was 30 mmHg. A focal whitish lesion was present at the foveal center (Figure 1C). OCT obtained using ZEISS CIRRUS HD-OCT demonstrated marked hyperreflectivity involving nearly the entire inner retina at the fovea, with posterior shadowing and disruption of the ellipsoid zone (Figure 1B). This pattern was broader than the middle retinal band expected in PAMM. Fluorescein angiography showed mild macular hyperfluorescence without apparent retinal vascular occlusion or retinal vasculitis (Figure 1D). Macular OCT angiography was performed using CIRRUS HD-OCT 5000 with AngioPlex (Carl Zeiss Meditec, Dublin, CA, USA) with a fovea-centered 6 x 6-mm scan. The superficial and deep retinal slabs were generated automatically using the built-in AngioPlex software. The segmentation boundaries and corresponding B-scans were reviewed for obvious segmentation errors, and no manual correction was required. The segmentation boundaries and corresponding B-scans were reviewed for obvious segmentation errors, and no manual correction was required. No detectable perfusion abnormality was identified within the scanned macular area in either slab (Figures 1E, 1F). Dilated peripheral retinal examination and widefield fundus photography showed no visible peripheral necrotizing lesion (Figure 2); scleral depression was not performed. Although the presentation was atypical, ARN was included in the differential diagnosis. Oral valacyclovir 500 mg twice daily and topical antiglaucoma medications were initiated.

At the first visit to our hospital, best-corrected visual acuity was 1.5 in the right eye and 0.2 in the left eye. IOP was 12 mmHg in the right eye and 15 mmHg in the left eye while the patient was receiving topical antiglaucoma therapy. Laser flare photometry was 6.7 photon counts per millisecond in the right eye and 19.2 photon counts per millisecond in the left eye. Slit-lamp examination of the left eye showed fine keratic precipitates without anterior chamber cells. Anterior vitreous cells and vitreous opacity were not observed. Dilated fundus examination and widefield imaging showed no visible peripheral whitish lesion, retinal hemorrhage, or apparent retinal vasculitis. Color fundus photography documented the foveal lesion (Figure 1H). Scleral depression was not performed. OCT using ZEISS CIRRUS HD-OCT showed persistent broad inner retinal hyperreflectivity with foveal ellipsoid zone disruption (Figure 1G). Aqueous humor was collected at this visit for a 24-target multiplex ocular pathogen PCR assay while no peripheral retinal lesion was clinically evident.

Four days after presentation to our hospital, dilated fundus examination and ultra-widefield imaging documented a new inferotemporal peripheral retinal lesion between the 4- and 5-o'clock positions. The lesion measured approximately four disc diameters and was accompanied by vascular sheathing, punctate retinal hemorrhages, and vitreous opacity. These findings were clinically consistent with necrotizing herpetic retinitis. On the same day, the multiplex PCR result from the aqueous sample obtained at the first visit became available and was positive only for VZV DNA. HSV-1, HSV-2, Epstein-Barr virus, cytomegalovirus, human herpesviruses 6, 7, and 8, and all other tested infectious targets were negative. These findings established VZV-associated ARN.

Oral valacyclovir, which had been started at 500 mg twice daily three days before presentation, was increased to 1000 mg three times daily on hospital day four. Oral betamethasone 2 mg/day was initiated on the same day after seven days of oral antiviral therapy. Intravenous acyclovir 750 mg three times daily was started on hospital day 10 and continued until day 25. Betamethasone was increased to 6 mg/day intravenously on day 11, reduced to 4 mg/day on day 18, and to 2 mg/day orally on day 25. From day 42, corticosteroid therapy was switched to prednisolone 10 mg/day, reduced to 5 mg/day on day 56, 2.5 mg/day on day 70, and 2.5 mg every other day on day 84, and discontinued on day 98. After completion of intravenous acyclovir, oral valacyclovir 1000 mg three times daily was resumed on day 25, reduced to 1000 mg twice daily on day 42 and 500 mg twice daily on day 56, and discontinued on day 70.

The peripheral lesion rapidly regressed, and the broad inner retinal hyperreflectivity gradually decreased. At three weeks, the peripheral lesion had resolved, and OCT showed partial restoration of the ellipsoid zone outside the foveal center (Figure 1I). At six weeks, best-corrected visual acuity improved to 0.5. At the final follow-up, 42 weeks after treatment initiation, best-corrected visual acuity had improved to 0.6, with no retinal detachment, recurrent retinitis, or involvement of the fellow eye (Figure 1J).

## Discussion

The main feature of this case was the appearance of peripheral acute retinal necrosis (ARN) after an initially isolated foveal lesion. Aqueous humor was obtained at our first examination, when no peripheral lesion was seen on dilated examination or widefield fundus photography. Four days later, peripheral necrotizing retinitis developed, and multiplex polymerase chain reaction (PCR) of the previously collected aqueous sample was positive only for Varicella-zoster virus (VZV) DNA. These findings suggest that the foveal lesion was an early manifestation of VZV-associated ARN rather than an unrelated macular disorder.

The optical coherence tomography (OCT) morphology should be distinguished from paracentral acute middle maculopathy (PAMM). PAMM is an ischemic OCT finding characterized by a placoid or band-like hyperreflective lesion centered in the outer plexiform and inner nuclear layers, often followed by thinning of the inner nuclear layer [10]. In this patient, the OCT abnormality involved nearly the entire inner retina, produced substantial posterior shadowing, and was accompanied by ellipsoid zone disruption. The lesion was also clinically visible as a whitish foveal focus in an eye with ocular hypertension, fine keratic precipitates, and increased aqueous flare. Anterior chamber cells, anterior vitreous cells, and vitreous opacity were absent at the first examination at our hospital. Taken together, the morphology and inflammatory findings were not typical of isolated PAMM. A focal macular ischemic process was initially considered because no peripheral lesion or angiographic vasculitis was documented; however, the subsequent clinical course and VZV-positive aqueous PCR strongly favored viral retinitis.

Macular or posterior pole involvement at the onset of ARN has been described previously [4-9]. These reports show that the first clinically apparent lesion may be located close to the fovea or within the posterior pole before peripheral necrosis becomes obvious. The present case provides serial multimodal imaging together with VZV-positive PCR findings from an aqueous sample collected before a peripheral lesion became visible. Widefield fundus photography at the referring hospital showed no visible peripheral lesion within the photographed field. However, the method of peripheral retinal examination at the first local ophthalmology clinic was not documented, and scleral depression was not performed at the referring hospital or at our first examination. Therefore, a very small far-peripheral lesion cannot be completely excluded.

Aqueous humor PCR was important in establishing the viral diagnosis. PCR testing is particularly useful when the clinical presentation is incomplete or atypical [12,13]. In this case, a 24-target multiplex panel detected VZV DNA alone in an aqueous sample collected before a peripheral lesion became visible. Herpes simplex virus and cytomegalovirus were negative, supporting VZV as the cause of the intraocular infection. The patient had already started oral valacyclovir before presentation to our hospital, which may have modified the subsequent clinical course. Early antiviral therapy may have contributed to the limited extent of retinal necrosis and the absence of retinal detachment during 42 weeks of follow-up.

The combination of ocular hypertension and subtle anterior uveitis was diagnostically important. Herpes simplex virus, VZV, and cytomegalovirus can cause hypertensive anterior uveitis with keratic precipitates, and these anterior segment findings may provide an early clue to herpetic disease [14,15]. In the present case, the intraocular pressure of 30 mmHg at the referring hospital, fine keratic precipitates, and increased aqueous flare were not typical of a purely ischemic macular lesion. These findings supported continued observation for viral retinitis and early antiviral treatment before peripheral ARN became visible.

Progressive outer retinal necrosis (PORN) was also considered because VZV retinitis can involve the posterior pole early. PORN usually occurs in severely immunocompromised patients and is characterized by multifocal deep or outer retinal necrosis with relatively little intraocular inflammation [16,17]. In this patient, HIV serology was negative, and no evidence of a systemic autoimmune or connective tissue disease, diabetes mellitus, or malignancy was found. Ocular hypertension, keratic precipitates, increased aqueous flare, and the subsequent development of peripheral necrotizing retinitis with vascular sheathing, punctate retinal hemorrhages, and vitreous opacity favored ARN over a classic PORN presentation.

This report has several limitations. First, it describes a single patient. Although VZV DNA was detected in aqueous humor collected before peripheral retinitis became visible, this does not prove with certainty that the foveal lesion itself was caused by VZV. Second, the method of peripheral retinal examination at the first local ophthalmology clinic was not documented. Dilated examination and widefield fundus photography at the referring hospital and our hospital showed no peripheral lesion, but scleral depression was not performed; therefore, a very small far-peripheral lesion cannot be completely excluded. Third, the earliest OCT image was obtained using a Topcon system, whereas subsequent OCT images were obtained using CIRRUS HD-OCT. Direct comparison between scans obtained using different platforms was therefore limited. Fourth, OCT angiography was limited to a 6 × 6-mm macular scan; vascular abnormalities outside the scanned area could not be assessed. Finally, valacyclovir started before presentation to our hospital may have altered the subsequent clinical course.

## Conclusions

This case suggests that VZV-associated ARN may initially present as an isolated foveal lesion before peripheral necrotizing retinitis becomes visible. Broad inner retinal hyperreflectivity with posterior shadowing is not typical of PAMM when the abnormality is not confined to the middle retinal layers and should prompt consideration of other diagnoses, including viral retinitis.

When a focal foveal white lesion is accompanied by ocular hypertension, keratic precipitates, or increased aqueous flare, herpetic retinitis should remain in the differential diagnosis even when peripheral necrosis is not visible and no perfusion abnormality is detected within the acquired 6 x 6 mm macular OCTA scan. Careful peripheral retinal surveillance, timely intraocular fluid PCR testing, and prompt antiviral therapy are important to limit progression and preserve vision.
